# iTRAQ Mitoproteome Analysis Reveals Mechanisms of Programmed Cell Death in *Arabidopsis thaliana* Induced by Ochratoxin A

**DOI:** 10.3390/toxins9050167

**Published:** 2017-05-18

**Authors:** Yan Wang, Xiaoli Peng, Zhuojun Yang, Weiwei Zhao, Wentao Xu, Junran Hao, Weihong Wu, Xiao Li Shen, Yunbo Luo, Kunlun Huang

**Affiliations:** 1 Beijing Advanced Innovation Center for Food Nutrition and Human Health, College of Food Science & Nutritional Engineering, China Agricultural University, Beijing 100083, China; xuwentao1111@sina.com (W.X.); lyb@cau.edu.cn (Y.L.); 2 Institute of Food Science and Technology, Chinese Academy of Agricultural Sciences, Beijing 100193, China; wangyan062006@163.com (Y.W.); 3 Beijing Laboratory for Food Quality and Safety, College of Food Science and Nutritional Engineering, China Agricultural University, Beijing 100083, China; pxlpxh@163.com (X.P.); yzjscu2006@163.com (Z.Y.); zwwlucky115@163.com (W.Z.); lemontree_2010@sina.com (J.H.); isfdalss@163.com (W.W.); xiaolishen1983@163.com (X.L.S.); 4 College of Food Science and Engineering, Northwest A&F University, Yangling 712100, China; 5 School of Public Health, Zunyi Medical University, Zunyi 563000, China

**Keywords:** ochratoxin A, *Arabidopsis thaliana*, programmed cell death, mitochondria, iTRAQ, mitoproteomes

## Abstract

Ochratoxin A (OTA) is one of the most common and dangerous mycotoxins in the world. Previous work indicated that OTA could elicit spontaneous HR-like lesions formation *Arabidopsis thaliana*, reactive oxygen species (ROS) play an important role in OTA toxicity, and their major endogenous source is mitochondria. However, there has been no evidence as to whether OTA induces directly PCD in plants until now. In this study, the presence of OTA in *Arabidopsis* *thaliana* leaves triggered accelerated respiration, increased production of mitochondrial ROS, the opening of ROS-dependent mitochondrial permeability transition pores and a decrease in mitochondrial membrane potential as well as the release of cytochrome c into the cytosol. There were 42 and 43 significantly differentially expressed proteins identified in response to exposure to OTA for 8 and 24 h, respectively, according to iTRAQ analysis. These proteins were mainly involved in perturbation of the mitochondrial electron transport chain, interfering with ATP synthesis and inducing PCD. Digital gene expression data at transcriptional level was consistent with the cell death induced by OTA being PCD. These results indicated that mitochondrial dysfunction was a prerequisite for OTA-induced PCD and the initiation and execution of PCD via a mitochondrial-mediated pathway.

## 1. Introduction

Programmed cell death (PCD) occurs throughout the life cycle of plants during the course of development and in response to various biotic and abiotic stresses. PCD can be distinguished from accidental cell death using morphological criteria including chromatin condensation, nucleus and cell fragmentation [1]. The process of PCD is extremely complex, and many factors are involved and, among them, the role of mitochondria is indispensable. Mitochondria are sometimes described as ‘cellular power plants’, since they generate most of the cellular supply of adenosine triphosphate (ATP), used as a source of chemical energy [2]. Striking evidence supports the importance of signals from mitochondria during the formative stage of PCD in plants [3,4,5]. The involvement of mitochondria in plant stress responses and plant PCD has also been demonstrated: mitochondrial oxidative bursts are involved in the PCD response in oats [6]; and oxidative stress firstly increased respiration and generation of reactive oxygen species (ROS), resulting in ATP depletion and opening of mitochondrial permeability transition pores (MPTPs), then PCD [7]. More recently, Wu et al. reported that high fluence low-power laser irradiation (HF-LPLI) induced PCD via the mitochondrial/caspase pathway, and the link between opening MPTPs and triggering ROS could be a fundamental phenomenon in HF-LPLI-induced PCD [8]. In addition, Sun et al. showed that menadione can induce PCD in tobacco protoplasts with concomitant leakage of cyt c from mitochondria [9]. In addition, Vacca et al. demonstrated that *cyt* c is released in a ROS-dependent mode and is degraded via caspase-like proteases in tobacco Bright-Yellow 2 cells en route to heat shock-induced cell death [10]. Additionally, Balk et al. showed that cyt c release is frequently observed prior to hallmark morphological features of PCD, but little evidence as yet that cyt c can induce PCD, while the intermembrane space of plant mitochondria contains a DNase activity that may be involved in PCD [11]. Mitochondrial structure and function are also altered significantly during the process [6,12]. These findings indicated the close relationship between mitochondria and PCD, and while multiple mitochondrial factors such as ROS, cyt c, MPTP, respiration, mitochondrial membrane potential (ΔΨm) and ATP participated in PCD, these factors contacted and interacted with one another, forming a complex network and then regulating PCD.

Ochratoxin A (OTA) is a mycotoxin produced by several kinds of fungi, such as *Aspergillus ochraceus*, *A. carbonarius*, *A. niger* and *Penicillium verrucosum* [13,14,15]. OTA is one of the most common and dangerous mycotoxins in the world. Recently, much attention in studies on the toxicity mechanism of OTA has focused on animal experiments and cytotoxicity due to the deleterious effect of the mycotoxin on human health, and it has been acknowledged that OTA has carcinogenic, nephrotoxic, teratogenic, neurotoxic and immunotoxic properties [16]. Moreover, apoptosis in response to OTA is well documented in human and animal tissue-culture cells [17,18]; and increased ROS, decreased ΔΨm and increased cell death induced by OTA were detected in human embryonic kidney 293 cells [19]. However, the hosts of the fungi that produce mycotoxins are mainly plants (cereal, grape or bean), and the effect of mycotoxins on human health are only a consequence of ingesting infected plants and are not the primary function of these compounds. What effect, if any, these compounds have on their plant host, and what ecological role the mycotoxins play remain complicated questions. Although OTA could elicit spontaneous HR-like lesions formation in *Arabidopsis thaliana* [20,21], there have been no evidence on whether OTA induces directly PCD in plants until now.

*A. thaliana* has been described as a model organism for a number of mycotoxins, including fumonisin B1 (FB1) [22,23], AAL toxin [24] and the trichothecene family of toxins [25,26]. In our previous study, hypersensitive response-type lesions were observed in excised leaves infiltrated with OTA, such as the occurrence of oxidative bursts, phenolic compounds (autofluorescence) and deposition of callose [20]. ROS play an important role in OTA toxicity, and their major endogenous source is mitochondria. ROS can activate antioxidant enzymes and excessive ROS can react with membrane lipids to generate malondialdehyde (MDA) [21,27,28]. Several morphological characteristics of PCD were observed in leaves infiltrated with OTA, including chromatin condensation and breaking of the nucleolus. In multicellular organisms, PCD processes are in part governed by complex variations in transcriptional activities. We applied the second Solexa/Illumina Genome Analyzer platform to perform digital gene expression (DGE) profiling analysis of the *Arabidopsis* transcriptome response to OTA stimuli [28]. Through gene ontology (GO) analysis, 164 and 337 genes were found to be differentially expressed in mitochondria and the cell nucleus, respectively. The DGE data were consistent with DNA degradation and deoxyribonuclease activities detected in other PCD systems, which support that the cell death induced by OTA in Arabidopsis is PCD. The differentially regulated genes associated with the TCA cycle and oxidative phosphorylation were associated with the changes in mitochondrial function, suggesting that mitochondria may be involved in OTA-induced PCD.

As described above, plant cells can respond to various stimuli by initiating PCD, and OTA can induce apoptosis in human and animal tissue culture cells. In this study, we investigated whether mitochondria was involved in the PCD induced by OTA in *A. thaliana* for the first time. Proteomics research approach paves the way for a better comprehending the OTA toxicity mechanisms, and isobaric tags for relative and absolute quantification (iTRAQ) technology with relatively high sensitivity and reproducibility features has gained great popularity in quantitative proteomics applications [19,29,30]. The aim of this manuscript was to apply an iTRAQ-based proteomics method to determine whether or not mitochondrial dysfunction is a prerequisite for OTA-induced PCD and the initiation and execution of PCD via a mitochondrial-mediated pathway.

## 2. Results

### 2.1. Effect of OTA on mtROS Content and Respiration Rate

**Figure 1:**
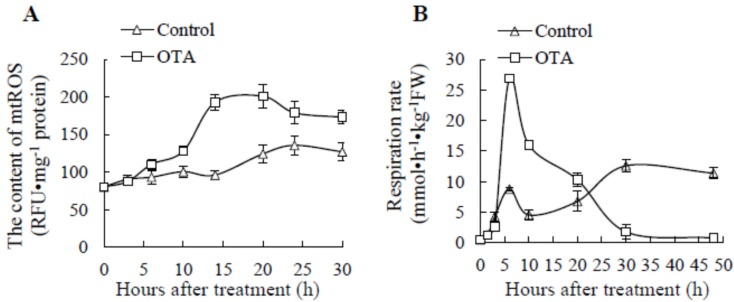
ROS production in isolated mitochondria of leaves (**A**) and the respiration intensity of *Arabidopsis* leaves (**B**) after treatment with 0.25 mM OTA. The mean value was calculated by three replicates, and the standard deviation was marked in the vertical bars.

Mitochondrial oxidative burst is involved in apoptotic response and ROS is considered a mediator of PCD [6]. ROS production in isolated mitochondria of *Arabidopsis* leaves increased both in control and OTA-treated samples (Figure 1A), and mtROS contents (including O_2_^.−^, H_2_O_2_ and OH^.^) in OTA-exposed leaves were significantly higher (*P* < 0.05) than in controls after 5 h, indicating that OTA effectively improved mtROS accumulation.

Cellular respiration is also known as ‘oxidative metabolism’ and the respiration rate of fresh produce can be expressed as O_2_ consumption rate [7,31] and/or CO_2_ production rate [32]. There was a remarkably sharp increase in respiration rate, measured as evolved CO_2_ by GC analysis, in mitochondria from leaves treated with 0.25 mM OTA for 3–10 h, reaching a maximum of 26.9 nmol h^−1^ kg^−1^ fresh weight at 6 h and declining dramatically in the later stage (Figure 1B). There was a relatively small peak at 6 h after the onset of treatment in the control groups, probably induced by the wound at the petiole.

### 2.2. Mitochondrial Swelling and Decreased ΔΨm in OTA Treatment

**Figure 2:**
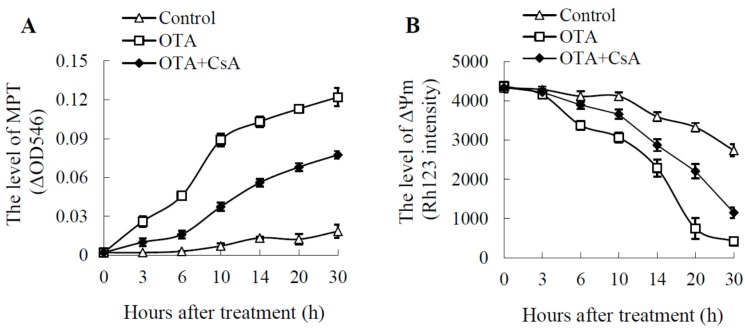
The effect of OTA stress on mitochondrial permeability transition (MPT) and mitochondrial membrane potential (ΔΨm). (**A**) MPT was measured as the difference in the change in OD_546_. Permeability transition was initiated by addition of 16.5 nM CaCl_2_, and the decrease in absorbance was measured at 546 nm. (**B**) The loss of electric potential of the mitochondrial membrane of *Arabidopsis* protoplasts stained by Rh123. The mean value was calculated by three replicates, and the standard deviation was marked in the vertical bars. OTA = 0.25 mM OTA, Control = equal volume methanol.

MPT is associated with PCD in a majority of cases from diverse stimuli [7]. Although mitochondria from control groups swelled considerably, the decrease of absorbance at 546 nm in treatment groups was increased significantly (Figure 2A), which means that the opening degree of MPTPs was increasing continuously. CsA, as a specific inhibitor of MPTP opening, it could be specifically combined with cyclophilin D, a MPTP matrix component, thereby inhibited the opening of MPTP and protected cells from death. The results showed that CsA could partially suppress the opening of MPTP induced by OTA (Figure 2A).

The uptake of Rh123 into mitochondrial matrix is dependent on MPT. Rh123 was chosen as a specific fluorescent probe to be used to compare ΔΨm between OTA-treated mitochondria of *Arabidopsis* leaves and the methanol control. Rh123 intensities in OTA-treated samples decreased markedly during the entire treatment time (Figure 2B). Compared with controls, Rh123 intensities were significantly lower at 6 h after treatment, and the intensity declined to 420.2, which was only 15.3% of control values at 30 h. The addition of CsA could slow down the decline of the ΔΨm induced by OTA (Figure 2B).

### 2.3. PCD-like Features in OTA-treated Arabidopsis Protoplasts

**Figure 3:**
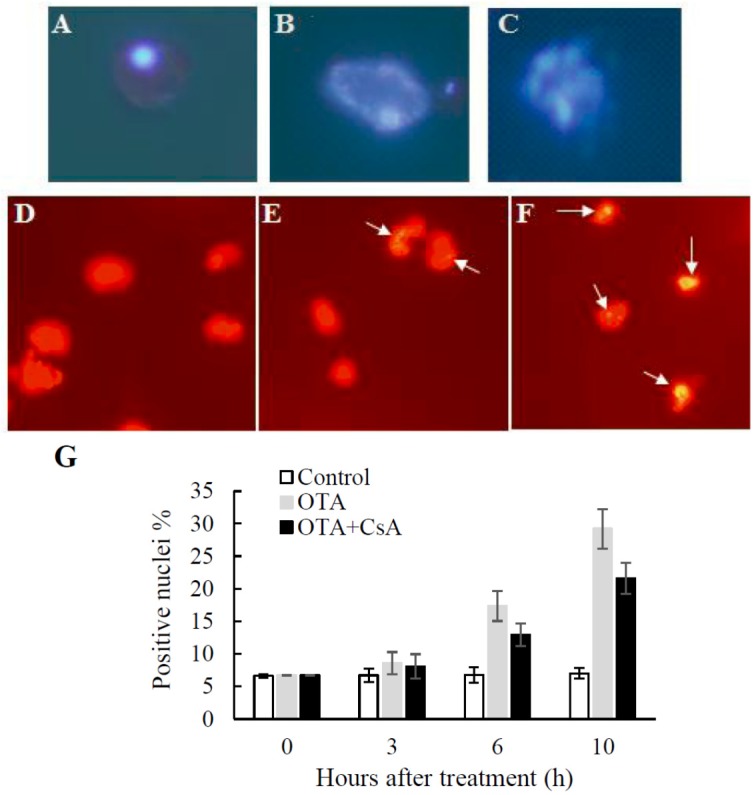
Detection of DNA fragmentaion in OTA-treated *Arabidopsis* protoplasts. (**A–C**) Nuclear apoptotic alterations in *Arabidopsis* protoplasts treated with 0.01 mM OTA for 10 h. Protoplasts labeled with the Hoechst 33342 (6.25 µg/mL) and observed under UV light excitation. (**A**) Control: Normal protoplast. (**B**) Cell deformed, swelled and chambered. (**C**) Chromatin condensed and apoptotic body formed (1000×). (**D–F**) Example of TUNEL-positive nuclei in OTA treated *Arabidopsis* protoplasts incubated with 0.01 mM OTA for 10 h. Protoplasts labeled with the TUNEL reaction observed under blue light excitation (**D**) Surviving protoplasts. (**E,F**) Protoplasts labeled with TUNEL reaction observed under blue light excitation. White arrow indicate TUNEL-positive nucleus (600×). (**G**) The percentage of TUNEL-positive nuclei in different treatment. For each replicate at least 100 protoplasts were evaluated. The mean value was calculated by three replicates, and the standard deviation was marked in the vertical bars.

In plants, the PCD process is featured by certain apoptotic hallmarks such as cell shrinkage, chromatin condensation and genomic DNA degradation [11]. Hoechst 33342 is a cell-permeable DNA that is excited by ultraviolet light and emits blue fluorescence and is used for specifically observing the morphology of nucleus. As illustrated in Figure 3A, the normal cells and nucleus presented relatively regular shape, while some protoplasts showed the hallmark characteristic changes, such as swelling, deformation, cavitation, chromatin condensation and margination (Figure 3B), and even some protoplasts nucleus treated with 0.01 mM OTA for 10 h collapsed into apoptotic bodies (Figure 3C).

The TUNEL reaction which labels the free 3′-OH DNA extremities and constitutes a marker of plant PCD is used to visualize in situ the DNA fragmentation occurring during PCD [1]. Cleavage of genomic DNA into oligonucleosomal fragments yields many single-strand breaks (nicks), then generate free 3′-OH terminal which can be labeled. Figure 3E,F showed typical results of the experiment, the nucleus labeled by TUNEL indicated the DNA breakage associated with PCD was taking place in these protoplasts treated with 0.01 mM OTA for 10 h. In contrast, there was little TUNEL staining in normal protoplasts, as shown in Figure 3D, indicating the nucleus of the protoplasts remain intact. Statistical analysis of TUNEL-positive nuclei is shown in Figure 3G. The number of TUNEL-positive nuclei at 6 h and 10 h after OTA treatment increased significantly by 2.58 and 3.41 fold compared to control. The results showed that CsA could reduce the number of TUNEL-positive nuclei at 6 h and 10 h significantly by 74.6% and 74.0% (Figure 3G). These data demonstrate that DNA fragmentation occurred in the OTA-treated Arabidopsis protoplasts.

### 2.4. Cyt c Release Occurs in OTA-induced PCD of Arabidopsis Leaves

**Figure 4:**
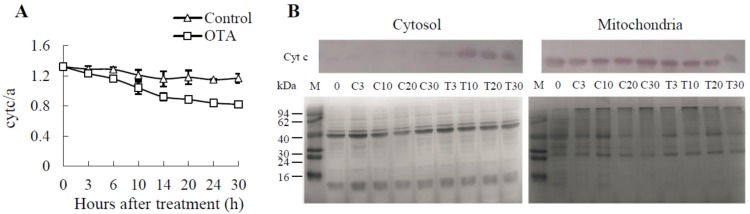
The effect of OTA on cyt c/a and the release of cyt c detected by western blot analysis. (**A**) The relative cyt c/a content in mitochondria was determined spectrophotometrically by the ratio of absorbances at 550 nm/603 nm (i.e., the maxima of absorbance for cyt c and a, respectively). Each value represents the mean of three replicates, and the vertical bars indicate the standard deviation. (**B**) Western blot analysis of distribution of cyt c in cytosol and mitochondria during OTA stressed *Arabidopsis* leaves. Immunoblot of a 15% SDS-PAGE gel probed with a monoclonal anti-cyt c antibody. Staining of mitochondrial and cytosol protein in electrophoresis map to reveal the amount of sample loaded. OTA = 0.25 mM OTA, Control = equal volume methanol. From left to right: Control: 0, 3, 10, 20 and 30 h; OTA: 3, 10, 20 and 30 h.

Cyt c is a relatively small soluble protein (about 13 kDa) loosely bound to the surface of the inner mitochondrial membrane, while cyt a is tightly attached to the internal membrane. Therefore, the relative cyt c/a content can reflect the content of cyt c in the inner mitochondrial membrane. In our model, during the first 3 h, the decrease of cyt c/a did not vary significantly; however, 6 h after the treatment, the ratios of cyt c/a were obviously lower in OTA-treated mitochondria than in controls (*P* < 0.05) (Figure 4A).

Cyt c release from mitochondria was further tracked by immunoblot analysis. Both cytosolic and mitochondrial fractions, obtained from *Arabidopsis* leaves subjected to 0.25 mM OTA, were examined. Typical immunoblots are shown in Figure 4B, there were no obvious signals in the control cytosol, while release of cyt c into cytosol from mitochondria was observed at 3 h in leaves exposed to OTA, and large amount of cyt c was detected after 10 h. The amount of cyt c in the corresponding mitochondrial fractions, evaluated in the same cellular preparation, decreased with respect to controls, indicating a release of cyt c from mitochondria to cytosol.

Taken together, the loss of MPT, the decrease of ΔΨm and the release of cyt c occurred at the early stage after OTA treatment, and CsA could partially suppress the opening of MPTP induced by OTA, that implied that mitochondrial dysfunction was involved in the process of OTA-induced PCD.

### 2.5. iTRAQ Analysis of OTA-Responsive Mitochondrial Proteins in Arabidopsis Leaves

Total mitochondrial proteins were prepared from *Arabidopsis* leaves for 8 and 24 h in Petri dishes already containing 0.25 mM OTA or the corresponding concentration of methanol, for the mitochondrion was relative intact when OTA treated for 8 h, and it was destroyed when OTA treatment extended to 24 h. An iTRAQ-based proteomics method was adopted to quantify the differentially expressed mitoproteome with OTA treatment. A total of 3226, 4410 and 3724 distinct peptides were identified from 46973, 70907 and 64510 spectra in three biological replicates, respectively. The corresponding number of identified proteins with unused score > 1.3 was 329, 396 and 336; among these proteins, the number of identified proteins with peptides ≥ 2 was 210, 257 and 210. In order to determinate the relative abundance of the proteins in each sample type, a selection parameter requiring the detection of any identified protein in at least two of the three iTRAQ experiments was applied. A total of 42 and 43 proteins met all four conditions (unused score > 1.3, 95% CI, more than one peptide detected, and detected in at least two of three experiments) and were used in subsequent quantitative analysis, respectively.

For assessment of variation in the iTRAQ quantification experiment, the ratio of differentially expressed proteins (*P* < 0.05) were compared in response to OTA versus control across at least two biological replicates against the number of peptides identified. The significance threshold was showed in a powerful visual indication accomplished through Whetton’s plot. Among 42 and 43 differentially expressed proteins identified from OTA treatment for 8 and 24 h, respectively, 39 and 41 proteins were within |log (ratio)| of 0.1, suggesting that variation from the interbiological replicates was 95%. In consequence, the proteins with log (ratio) of >0.1 or <−0.1, which correspond to relative expression of >1.26 or <0.79 (95% confidence), were chosen for further analysis (Table 1).

### 2.6. Classification of Differentially Expressed Mitoproteome

**Figure 5:**
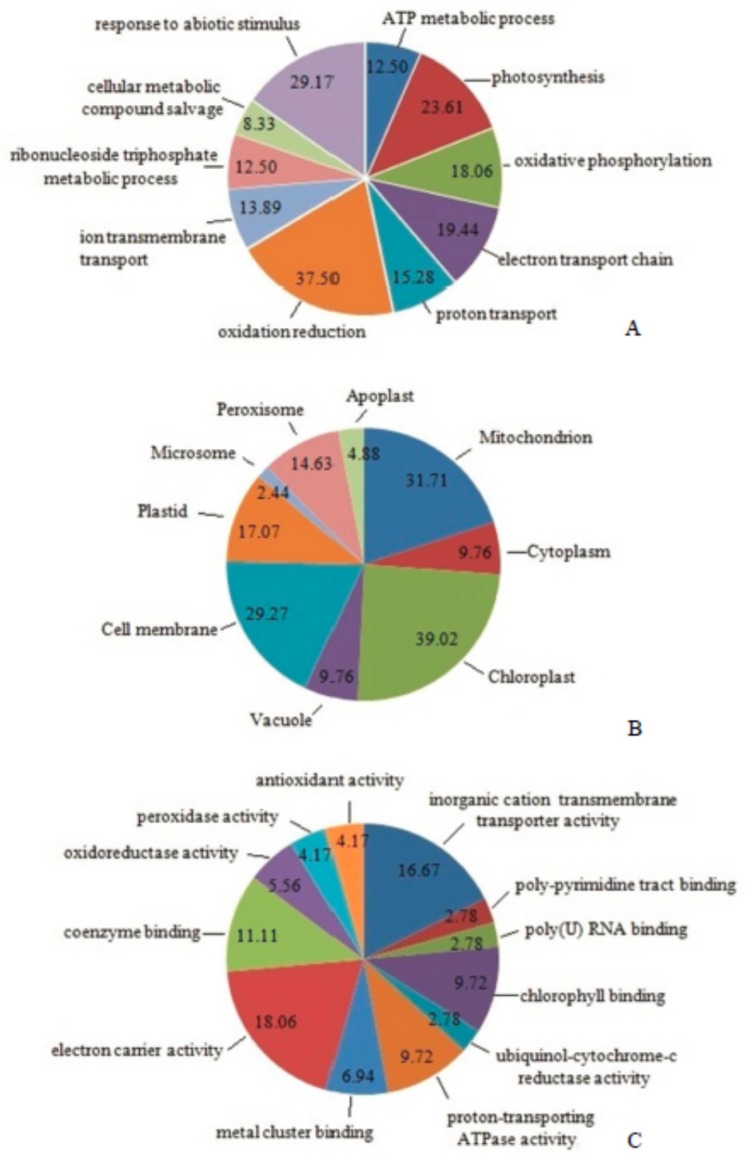
The differentially expressed mitochondrial proteins in response to OTA quantified by iTRAQ were classified according to Gene Ontology (GO): biological processes (**A**), cell components (**B**) and molecular functions (**C**) using the David database.

The identified differentially expressed proteins were functionally catalogued according to Gene Ontology (GO) biological processes (Figure 5A), cellular components (Figure 5B) and molecular functions (Figure 5C) using the David database. Proteins were involved in such biological processes as ATP metabolic process (12.50%), photosynthesis (23.61%), oxidative phosphorylation (18.06%), electron transport chain (19.44%), proton transport (12.50%), oxidation-reduction (37.50%), ion transmembrane transport (13.89%), ribonucleoside triphosphate metabolic process (12.50%), cellular metabolic compound salvage (12.50%) and response to abiotic stimulus (29.17%) (Figure 5A). Reported in the research of Millar et al., approximately 48% of proteins were separated in *Arabidopsis* mitochondrial proteome [33]. Here 43.50% of the identified proteins and 31.71% of the differential expression proteins were located in mitochondria in iTRAQ-based mitoproteome (Figure 5B). Molecular function of the identified proteins was classified into: electron carrier activity (18.06%), inorganic cation transmembrane transporter activity (16.67%), coenzyme binding (11.11%), proton-transporting ATPase activity (9.72%), metal cluster binding (6.94%), oxidoreductase activity (5.56%), peroxidase activity (4.17%), antioxidant activity (4.17%) and ubiquinol-cytochrome-c reductase activity (2.78%) (Figure 5C).

The differentially expressed proteins were classified into five groups by integrating GO and PANTHER biological processes, molecular functions, or KEGG pathways with the STRING database (Table 2). Each protein-protein interaction network, including photosynthesis, mitochondrial electron transport, ATP synthesis, protein biosynthesis and mitochondrial transport protein are shown in Figure 5A. The protein-protein interaction of 23 differential expression proteins located in mitochondria was analyzed in the STRING database (Figure S1).

The differentially expressed proteins involved in photosynthesis (Group 1) were all located in chloroplast (Table 2)—all proteins were downregulated except for oxygen—evolving enhancer protein (Q9XFT3), which was upregulated at 24 h of treatment, indicating that photosynthetic capability of leaves treated by OTA had declined. Five proteins (Q9FGI6, Q9SUU5, Q9FLX7, O82663 and O22769) involved in mitochondrial electron transport (Group 2) were upregulated by OTA treatment for 8 and 24 h. The electron transport respiratory chain of mitochondria couples the oxidation and phosphorylation of ADP to form ATP, and provides energy for numerous biological processes. The expression of proteins (Q8W4E2, Q9FT52 and O82628) related to ATP synthesis (Group 3) were increased by 8 h of treatment, except for ATP synthase subunit b (P56759). With increasing treatment duration (24 h), all identified proteins (O23654, P56757, P09468, Q01908, P19366, Q39258 and O82628) related to ATP synthesis were downregulated. Other than 60S ribosomal protein L13-1 (P41127), all proteins (P51413, P56793, Q9LXG1, Q9FDZ9 and P51418) related to ribosome biogenesis (Group 4) were downregulated, indicating that OTA inhibited nucleotide translation and then weakened DNA repair. Mitochondrial membrane binding proteins (Group 5), including voltage dependent anion channel (VDAC, Q9FJX3, Q9SRH5 and Q9SMX3) increased significantly, suggesting that the mitochondrial membrane integrity was damaged and the membrane permeability increased.

### 2.7. DGE Profiling Analysis

Since mitochondrial dysfunction and DNA damage were involved in the process of OTA-induced PCD, as indicated by the changes in physiological-biochemical characteristics, here we mainly focused on genes involved in mitochondrial pathway and nucleic acid metabolism detected in DGE analysis. The nucleotide samples were extracted from leaves treated with 0.25 mM OTA and methanol control for 8 h. The complete DGE data were published as a supplement in Wang et al. [21].

### 2.8. The Expression Profile of Nucleic Acid Metabolism

The DGE data suggested that endonuclease was responsible for DNA damage, DNA damage was associated with endonuclease in a majority of cases, nine deoxyribonucleic acid genes were induced by OTA exposure in the DGE data. Moreover, four of them were induced >50-fold: demeter-like 2, chlororespiratory reduction 28, endonuclease and essential meiotic endonuclease 1A (Table 3). The differentially expressed genes related to endonuclease presented here were consistent with the morphological analysis that nuclear DNA degradation was detected by TUNEL assay (Figure 3), a known marker of PCD.

In contrast to the elevation of genes responsible for DNA fragmentation, the expression of most genes (80%) encoding DNA replicate proteins decreased (Table 3). Conversely, two genes (AT1G10590 and AT4G17020) engaged in DNA repair were upregulated 2.2- and 2.1-fold, respectively (Table 3).

All the differentially expressed genes encoding RNA polymerase that were detected in leaves exposed to OTA were upregulated (Table 3). It may be that PCD is a positive physiological cell-suicide process and that the organism requires RNA polymerase activity to regulate OTA-induced PCD. Two RNA degradation-related genes (AT2G36530 and AT1G17980) were induced by OTA exposure.

### 2.9. The Expression Profile of Oxidative Phosphorylation and Citric Acid Cycle in Mitochondria

A considerable fraction of the differentially regulated genes were associated with the TCA cycle and oxidative phosphorylation (Table 4). Among them, all eight differentially expressed genes encoding four enzymes (pyruvate dehydrogenase, citrate synthase, isocitrate dehydrogenase and 2-oxoacid dehydrogenase) that catalyze the rate-limiting steps of TCA were upregulated. Other differentially expressed genes encoding proteins, include aconitase, succinyl-CoA ligase (putative), fumarase and succinate dehydrogenase were upregulated, and only one gene encoding malate dehydrogenase was suppressed under OTA stress.

Along with the increased respiration rate, as shown by the upregulated genes of TCA and the increasing CO_2_ evolution rate (Figure 1B), oxidative phosphorylation in mitochondria seemed to be accelerated. A total of 19 genes coding for mitochondrial inner membrane proteins were differentially expressed - among these, 17 genes (89.5%) were positively associated with OTA and only two with opposite expression in OTA stimuli compared to control (Table 4). The transcript results were in close agreement with those obtained by the increase of respiration rate relative to the control treatment around 8 h timepoint as evaluated by CO_2_ evolution. The acceleration of electron transfer and more electrons leaking out of mitochondria membrane caused the increased content of ROS in mitochondria (Figure 1A).

## 3. Discussion

### 3.1. OTA Disturbed the Mitochondrial Electron Transport Chain

In plant tissue, ROS are considered as important signals in the activation of PCD. The addition of appropriate concentrations of exogenous ROS or ROS generators in tobacco Bright-Yellow 2 cells activated a process of death with the typical cytological and biochemical features of PCD [34]. Various conditions such as ozone fumigation, cold stress, UV radiation and senescence as well as hypersensitive response to pathogen attack lead to accelerated generation and/or accumulation of ROS and subsequent PCD [35,36]. In our experiments, the whole ROS amount quantitatively monitored by H_2_DCFDA probe in leaf tissue [20,21] and mitochondria (Figure 1A), were increased after OTA treatment.

Mitochondria are also one of the major sites producing ROS in plants. When electrons pass through complexes I–IV of the electron transport chain, 1–5% of electrons leak out and interact with oxygen to form superoxide [37]. Tiwari et al. demonstrated that the addition of complex I or III inhibitors diminished accumulation of H_2_O_2_ [7]. The ROS content in mitochondria was elevated significantly after 5 h in OTA-exposed leaves compared to control (Figure 1A). Eighty percent of differentially expressed complex I genes, and all of the differentially expressed complex III and IV genes, were upregulated at 8 h after treatment in the transcript analysis (Table 4). Three energy-transducing enzymes, including NADH: ubiquinone oxidoreductase (complex I), Coenzyme Q–cytochrome c reductase (complex III) and cytochrome c oxidase (complex IV) were involved in the electron transport chain [38]. Complex I might be the major site of superoxide production within mitochondria in the process of reverse electron transfer, with up to 5% of electrons being diverted to superoxide formation. The process by which electrons from the reduced ubiquinol pool (supplied by succinate dehydrogenase and glycerol-3-phosphate dehydrogenase) pass through complex I to reduce NAD^+^ to NADH is driven by the inner mitochondrial membrane potential electric potential [39,40]. Of the OTA-induced differential expression proteins identified by iTRAQ analysis, the expression level of NADH dehydrogenase (Q9FGI6, O22769 and Q9FLX7) increased by 1.357, 1.667 and 1.388 times, respectively; succinate dehydrogenase (O82663) was upregulated 1.305 times (Table 2); and glyceraldehyde-3-phosphate dehydrogenase (P25858) increased by 1.342 and 1.385 times after 8 h and 24 h, respectively (Table 2). Our results implied that during the process of reverse electron transfer, the mitochondrial complex I might be the most important site of ROS production induced by OTA; however, this requires further verification. Beyond that, chloroplast is another site of intercellular ROS production.

### 3.2. OTA Interferes with ATP Synthesis

Most proteins related to photosynthesis were downregulated in response to OTA according to iTRAQ analysis (Table 2), consistent with a previous report that OTA inhibited photosynthesis [28], with the result that OTA reduced ATP production during photoreaction. In order to maintain the energy requirement, mitochondrial respiration is accelerated to generate more energy. Most of the proteins related to ATP synthesis were upregulated by 8 h of OTA treatment (Table 2), and transcript analysis showed the same result (Table 4). Complex III played a key role in ATP formation (oxidative phosphorylation), and the level of cytochrome b–c1 complex (Q9SUU5) was upregulated 1.42 times by 8 h of OTA treatment (Table 2), indicating enhanced oxidative phosphorylation.

Meanwhile, the acceleration of respiration and oxidative phosphorylation would result in more leakage of electrons out of the mitochondrial membrane. Under such conditions, the generation and accumulation of ROS would increase, and may lead to irreversible oxidative damage of mitochondrial membrane proteins and lipids, and the opening of MPTP (Figure 2A), a decrease in ΔΨm (Figure 2B) and release of cyt c into the cytosol (Figure 4). When ΔΨm is lost, protons and some molecules can flow across the outer mitochondrial membrane uninhibited [41]. Loss of ΔΨm can interfere with ATP production, due the fact that an electrochemical gradient is needed to provide the driving force for ATP production in mitochondria. As a highly conserved protein across plants, animals and many unicellular organisms, cyt c transfers electrons between complex III and IV [42]. Therefore, the loss of cyt c, as a component of the electron transport chain, should block electron flow from complex III to IV and lead to uncoupling between oxidative (respiratory chain) and phosphorylation (ATP synthase), thus generating a very high level of ROS. Meanwhile, the expression level of ATP synthase identified by iTRAQ decreased after 24 h of OTA treatment (Table 2).

### 3.3. OTA Induces PCD in Arabidopsis thaliana

Apoptosis in response to OTA is well documented in human and animal tissue culture cells [17,18,19]. Our previous studies showed that OTA elicited HR-like lesion formation in *Arabidopsis* leaves, and the morphological characteristics were changed, including separation of the plasma membrane from the cell wall, chromatin condensation and the margination and breaking of the nucleolus, destruction of mitochondrial structure and escape of the mitochondrial matrix from mitochondria [21]. In situ TUNEL staining showed a representative TUNEL-positive cell (Figure 3E,F). The upregulated endonuclease, the inhibition of DNA replication and the active DNA repair were reflected in the transcript analysis (Table 3). This was consistent with DNA degradation and deoxyribonuclease activities detected in other PCD systems [43,44]. Thus, the result was consistency with the DNA fragmentation observed in TUNEL staining, which indicated that PCD was triggered by OTA in *Arabidopsis thaliana.*

The release of cyt c from mitochondria, one commitment step in signaling of apoptosis in animal cells, was found to be dispensable in PCD [45]. Western blot analysis demonstrated that OTA-induced cell death was also associated with cyt c release into the cytosol as early as 3 h after treatment (i.e., preceding cell death; Figure 4B). Cyt c releases from mitochondrion into cytosol and activates specific caspase-like enzymes in PCD triggered in the tomato fruit heat stress response [46]. This indicated that PCD induced by OTA mainly occurred early in the treatment. Additionally, the level of ΔΨm was significantly lower at 6 h after OTA treatment (Figure 2), and nucleus DNA breakage was detected at 6 h after OTA treatment by TUNEL (Figure 3). CsA could partially suppress the opening of MPTP induced by OTA (Figure 2A) and weaken the extent of OTA-induced cell death (Figure 3G). The release of cyt c and the loss of MPT happened at the early stage of PCD implies that the mitochondrial dysfunction may trigger PCD during OTA treatment.

VDAC was closely linked with mitochondrial processing peptidase (Q42290, AT1G51980) and ATP synthase (Q9SJ12, AT2G21870) (Figure S1). iTRAQ analysis showed that the levels of voltage dependent anion channel proteins VDAC1 (Q9SRH5), VDAC2 (Q9FJX3) and VDAC3 (Q9SMX3) all increased (Table 2). VDAC is considered an essential component of the MPTP, and studies have also identified a close correlation between inhibition of MPTP opening and phosphate transport into the mitochondria [47]. In mammalian cell, opening of the MPTP causes uncoupling of the mitochondria and swelling of the matrix, leading to rupture of the outer mitochondrial membrane and ultimately cell death [48]. VDAC1-dependent MPT engages a positive feedback loop involving mtROS (Figure 1A), and secondarily triggers a canonical apoptotic response including cyt c release (Figure 4) and caspase 3 activation. VDAC1-dependent MPT is an upstream mechanism playing a causal role in oxidative stress-induced apoptosis in tumor cells [49]. The high levels of ATP may contribute to stabilizing the tertiary structure of VDAC1, while shielding from pro-apoptotic factor binding, thus protecting tumor cells in a synergic way from PCD [50]. iTRAQ showed that ATP synthase and ADP, ATP carrier protein (P31167) were downregulated (Table 3) after 24 h of OTA treatment, indicating that the capability of ATP synthesis decreased with increasing duration of OTA treatment. Then the tertiary structure of VDAC1 changed and VDAC1-dependent MPT was lost, an event marking the point of no return in multiple pathways leading to cell death.

Overall, mtROS accumulation increased, loss of MPT and cyt c released from mitochondria into the cytosol happened at the early stage of OTA-induced PCD. This was followed by oxidative phosphorylation uncoupling, leading to disorder of respiration and stimulating mtROS rapid generation, thus amplifying the signaling cascade and terminating in PCD. The behavior of mitochondria further supports that the mitochondria dysfunction was a prerequisite for OTA-induced PCD and the initiation and execution of PCD via a mitochondrial-mediated pathway in *Arabidopsis thaliana*.

## 4. Experimental Section

### 4.1. Plant Material, Growth Conditions and OTA Treatments

Plants of *Arabidopsis thaliana* ecotype Columbia Col-0 were planted in soil 7 d after germinating on Murashige and Skoog medium under conditions (temperature of 20 ± 2 °C, relative humidity of 40–60%, 16/8 h of light/dark and photosynthetic photo flux density of 100 µE m^−2^ s^−1^). Four- to six-week-old plants were carried out the infiltration treatments. Briefly, excised leaves were incubated in Petri dishes containing 0.25 mM OTA or the corresponding concentration of methanol, which was used as a control as described previously [28]. After removed from OTA solution, leaves were quickly frozen in liquid N_2_ and then to biochemical measurements and mitochondria extraction.

### 4.2. Mitochondria Isolation

Mitochondria were isolated from *Arabidopsis* leaves by differential and sucrose density-gradient centrifugation as described by Ni et al. [51] and Millar et al. [33], with some modification. Briefly, leaves weighing about 14 g were ground in 70 mL of extracting solution 0.3 M mannitol, 50 mM sodium pyrophosphate, 0.5% [*w*/*v*] polyvinylpyrrolidone-40 (PVP-40), 0.5% [*w*/*v*] bovine serum albumin (BSA), 20 mM Cys and 2 mM EGTA at pH 8.0 and then passed through 50 µm nickel mesh. The filtered extract was initially centrifuged at 2000 *g* for 10 min at 4 °C and then the supernatant was centrifuged at 18,000 *g* for 15 min at 4 °C. The final organelle pellet was resuspended in 1 mL of resuspension buffer: 0.3 M mannitol, 0.1% [*w*/*v*] BSA and 20 mM TES (*N*-tris[hydroxymethyl]-methyl-2-aminoethanesulfonic acid)-NaOH at pH 7.5. About 2 mL of organelle suspension was layered on top of a sucrose gradient containing 6 mL of 0.6 M sucrose, 4 mL of 0.9 M, 6 mL of 1.2 M, 6 mL of 1.45 M and 6 mL of 1.8 M. The gradient was spun at 80 000 *g* for 90 min. About 300 µL of the mitochondrial fraction recovered from the 1.2 to 1.45 M sucrose density gradient were diluted fivefold with resuspension buffer (mentioned above) and centrifuged at 18 000 *g* for 15 min to precipitate the mitochondrial fraction.

The fractions were collected and measured the activities of marker enzymes using standard colorimetric methods, including cytochrome *c* oxidase for mitochondria, inosine 5′-diphosphatase for Golgi apparatus, catalase for peroxisomes and chlorophyll for chloroplasts.

### 4.3. Mitochondrial ROS (mtROS) Measurement

mtROS production was assessed using 2′,7′-dichlorofluorescein diacetate (H_2_DCF-DA), which detects ROS content, as described by Peng et al. [25].

### 4.4. Assessment of Mitochondrial Permeability Transition (MPT)

The opening of MPTPs causes mitochondrial swelling that is conveniently measured spectrophotometrically by recording the decrease in absorbance at 546 nm (OD_546_) [8]. In brief, freshly isolated mitochondria (0.3 mg protein mL^−1^) were suspended in respiration buffer containing 400 mM mannitol, 10 mM phosphate buffer (pH 7.5) and 1 mM EDTA. Then, 1 mM glutamic acid and 1 mM malate were added as respiratory substrates. OD_546_ was monitored at 25 °C for 10 min after the addition of 16.5 nM CaCl_2_ to initiate MPT. Swelling was inhibited by the addition of 1 µM cyclosporin A (CsA) as a reference to calculate the change in absorbance.

### 4.5. Measurement of Mitochondrial Membrane Potential (ΔΨm)

The ΔΨm was estimated by measuring the fluorescence of Rhodamine 123 (Rh123). Mitochondria (0.3 mg protein mL^−1^) suspended in incubation medium containing 20 mM Hepes-Tris pH 7.5, 0.4 M sucrose, 5 mM KH_2_PO_4_, 5 mM MgCl_2_ and 6 mM sodium succinate were incubated with Rh 123 at a final concentration of 2 µg·mL^−1^. After incubating the mixture at 25 °C for 30 min, the fluorescence was measured using GENios Pro Multifunction Microplate Reader (Tecan, Männedorf, Switzerland) with excitation at 485 nm and emission at 535 nm. The results were expressed as relative fluorescence mg^−1^ of protein.

### 4.6. Respiration Assay

CO_2_ emission was quantified by placing 10 washed leaves in a 20-mL airtight vial with 1 mL of water. After 3 h of incubation, a 1-mL sample was analyzed using a Shimadzu^TM^ 7840 gas chromatograph (Shimadzu, Tokyo, Japan), equipped with a CO_2_ conversion furnace, a Porapak Q column (80–100 mesh, 2 m × 3 mm) and a flame ionization (FID) detector. The carrier gas was N_2_ at a flow rate of 20 mL min^−1^, and the column, injector and detector temperatures were 80, 120 and 360 °C, respectively. To avoid modifications in the headspace gas composition due to gas sampling, each package was used for only one determination of the headspace gas composition.

### 4.7. Nuclear Morphology and DNA Fragmentation

Fresh isolated protoplasts were treated with 0.01 mM OTA or methanol control in culture media. Total DNA was visualized by staining with Hoechst 33342 (Sigma-Aldrich, St. Louis, MO, USA) at 6.25 µg/mL for 5 min at dark, and then visualized with an Olympus BX51 fluorescent microscope, using an UV filter.

For in situ detection of DNA fragmentation, the free 3’-OH groups in the DNA were labeled by the TUNEL (TdT-mediated-UTP-digoxigenin nick end labling) method, using a commercially available TUNEL kit (in situ cell death detection kit, Roche, Basel, Switzerland) as instructed by the manufacturer.

### 4.8. Measurement of Cytochrome c/a Ratio

The relative content of cytochrome *c*/*a* (cyt *c*/*a*) in mitochondria was measured by registration of differential spectra of absorbance of cytochromes [52]. Newly isolated mitochondria was suspended in 0.2 mg mL^−1^ BSA (0.3 mg mL^−1^ mitochondria protein mL^−1^), and the relative cyt *c*/*a* content was determined by the ratio between absorbances at 550/603 nm (i.e., the maxima of absorbance for cytochromes c and a, respectively).

### 4.9. Cyt c Western Blot

Mitochondria were separated with mitochondria extracting solution without BSA and filtered through 50-µm nickel mesh. Following the second centrifugation at 2000 *g* and 12 000 *g*, the supernatant thus obtained was taken to represent the cytosol fraction, and the pellet was resuspended in grinding buffer to represent the mitochondrial fraction. Of protein, 18 µg was separated on 15% SDS-PAGE gel. Separated proteins were transferred onto Immobilon-PVDF membrane in transfer buffer (25 mM Tris, pH 8.3, 192 mM glycine and 20% methanol). After blocking with 2% BSA, the membrane was incubated with a primary anti-cytochrome c monoclonal antibody (1:1000 dilution, 7H8.2C12, BD Pharmingen) for 2 h. Subsequently, the membrane was washed twice more and incubated with appropriate secondary goat anti-mouse antibody conjugated with horseradish peroxidase (1:1000 dilution, DakoCytomation) for 2 h. Then the membrane was washed and the chromogenic substrate BCIP/NBT (Amresco, Solon, OH, USA) was added to localize antibody binding.

### 4.10. iTRAQ Labeling and Mass Spectrometry Identification

The mitochondrial fractions were dissolved in iTRAQ buffer (Applied Biosystems, Foster, CA, USA). The protein concentration was determined by the Bradford method (Bio-Rad). Samples for iTRAQ analysis were collected at 8 h and 24 after 0.25 mM OTA incubation (OTA) and the control was treated with equal methanol (CK). Three replicates were prepared from three independent experiments of samples grown at different times.

iTRAQ labeling and mass spectrometry identification were carried out as described by Shen et al. with a minor revision [19]. Mitochondrial proteins were labeled by iTRAQ tags according to the manual of ABSciex [53]. In brief, mitochondrial proteins (100 µg) of each sample was mixed with 4-plex iTRAQ labeling, with three biological replicates for each experimental group. Individual protein samples were treated as follows: each protein pellet for four samples was first dissolved in 20 µL of dissolution buffer, denatured with 1 µL of denaturant, reduced with 2 µL of reducing reagent and incubated at 60 °C for 1 h, then alkylated with 1 µL of cysteine blocking reagent and incubated at room temperature for 10 min. Trypsin digestion occurred by adding 1 µL of 0.6 µg µL^−1^ Sequencing Grade Modified Trypsin (Promega, Madison, WI, USA) to each sample and incubating the samples at 37 °C for 16 h. The digested samples for groups CK (8 h), OTA (8 h), CK (24 h) and OTA (24 h) were labeled with iTRAQ tags 114, 115, 116 and 117, respectively (the iTRAQ tags were previously dissolved in 70 µL of ethanol individually). Four tagged samples were adjusted to pH 7.5–8.5 with dissolution buffer, placed at room temperature for 2 h and then merged together and lyophilized to powder.

The following step was used to remove the hydrolyzed unbound iTRAQ reagents in samples. The dried labeled samples were dissolved in 200 µL of buffer A [10 mM K_3_PO_4_, 5% acetonitrile (ACN), pH 2.5, with phosphoric acid] and loaded into a PolySULFOETHYL A column (200 mm length × 4.6 mm id, 200 Å pore size and 5 µm particle size) (PolyLC, Columbia, MD, USA). The sample was washed with 100% buffer A for 10 min at 40 µL min^−1^ to remove excess reagent and then eluted using 100% buffer B (10 mM K_3_PO_4_, 500 mM KCl, 25% ACN, pH 2.5, with phosphoric acid) for 5 min at 40 µL min^−1^. The eluent was collected and lyophilized to powder. Then the powder was dissolved in 400 µL buffer C (1% ACN and 0.1% TFA) and desalted through a Sep-Pak C18 cartridge (Waters, Milford, MA, USA). The desalted sample was then lyophilized to powder [54].

The desalted powder was dissolved in 100 µL mobile phase A (20 mM ammonium formate, pH 10.0, with ammonia water) and loaded into a Sepax poly high pH C18 column (2.1 mm id, 200 Å pore size and 5 µm particle size) on a LC-20AD system (Shimadzu, Tokyo, Japan). The sample was eluted using 5% to 35% mobile phase B (20 mM ammonium formate, 90% ACN, pH 10.0, with ammonia water) for 30 min, a gradient of 35–80% B for 2 min, 80% B for 3 min, 80% to 5% B for 1 min, and 5% B for 14 min, at 200 µL min^−1^ for total 50 min. The eluted fractions were monitored at wavelength of 214 nm. Fractions were collected every 2 minutes, and consecutive fractions with low peak intensity were pooled together. In total, 15 fractions were collected and lyophilized. Each dried fraction was reconstituted in 51 µL mobile phase A (5% ACN, 0.1% formic acid, pH 2.5, with formic acid), then loaded onto a CapTrap C18 column (500 µm × 2.0 mm) (Michrom Bioresources, Auburn, CA, USA) at a flow of 20 µL min^−1^. Peptide separation was carried out using a Nano Magic C18 column (15 cm × 100 µm, 200 Å pore size and 3 µm particle size) (Michrom Bioresources, Auburn, CA, USA). Mobile phases A and B (90% ACN, 0.1% formic acid, pH 2.5, with formic acid) were used to establish a 120-min gradient (5% B to 40% B for 100 min, up to 80% B for 1 min and 80% B for 4 min), and re-equilibrated at 5% B for 15 min finally. The parameters of Nano Aquity UPLC system (Waters, Milford, MA, USA) was set at 500 nL min^−1^ flow rate and 35 °Ccolumn temperature [29,30]. The electrospray voltage of the mass spectrometer and the heated capillary temperature was set to 1.8 kV and 200 °C. The data-dependent mode to switch automatically between MS and MS/MS acquisition was selected in the LTQ Orbitrap XL mass spectrometer (Thermo Fisher Scientific, Waltham, MA, USA) [30]. The centroid-scan (*m*/*z* 400–1800) was acquired in the Orbitrap with a mass resolution of 30 000 (*m*/*z* 400), followed by six sequential HCD (Higher energy C-trap dissociation)-MS/MS scans with a mass resolution of 7500. MS/MS analysis was operated on the top six most intense ions in centroid-scan mode with ion intensity > 5000, LTQ ion maximum IT of 500 ms, AGC of 2 × 10^5^ and with a precursor ion of 3 Da. For MS/MS, precursor ions were activated with 40% normalized collision energy and an activation time of 40 ms, with a charge state of ≥2^+^. The exclusion window was set 10 ppm ahead of the precursor ion m/z and 30 ppm behind it, and dynamic exclusion parameters were set to two repeat counts at 10-s repeat duration, and an exclusion list size of 180 s.

### 4.11. Database Identification and iTRAQ Quantification

The identification and quantification of raw peptide and protein were carried out using ProteinPilot v4.0 (Applied Biosystems, Foster, CA, USA) with the Paragon Algorithm against the UniProtKB/Swiss-Prot database (Release 2012_11, taxonomy: *Arabidopsis thaliana*, including 11571 sequences). Data search parameters were set as follows: trypsin cleavage with two missed cleavage; fixed modification of cysteines by methyl methanethiosulfonate (MMTS); and iTRAQ modification of peptide N termini, methionine oxidation and variable modification of lysine residues [19]. To reduce false positive identifications, unused score > 1.3 (equivalent to 95% confidence) and false discovery rate (FDR) < 1% were required for all identified proteins. For quantitative analysis, a protein must have a minimum of two unique peptide matches with iTRAQ ratios [54].

### 4.12. Mitoproteome Analysis

Differential expression proteins were classified according to annotations from the UniProt knowledge base (Swiss-Prot/TrEMBL, 24h) and the GO database. The biological process (BP) and molecular function (MF) were categorized using David database [55], GOTERM and PANTHER [56]. Cellular components (CC) were classified by GOTERM. Pathways were elucidated through KEGG_PATHWAY, PANTHER_PATHWAY and REACTOME_PATHWAY associated with the differentially expressed protein [55]. The protein-protein interaction networks among differentially expressed proteins were predicted and visualized using STRING database (version 9.0).

### 4.13. DGE Tag Profiling

The DGE profiling analysis was performed according to Wang et al. [21]. The results were considered to be statistically significant at *P* < 0.05.

### 4.14. Statistical Analysis

The statistical analyses were performed by Microsoft Excel 2007 and SPSS 13.0. Data were subjected to variance analysis (ANOVA), and means comparison was analyzed through Duncan’s multiple-range test. Differences were considered to be significant at *P* < 0.05.

## Figures and Tables

**Table 1:** The differentially expressed proteins in response to OTA treatment as quantified by iTRAQ.

Protein Name	UniProtKB Accession	Gene Names	Unused Protein Score	% of Coverage	No. of Unique Peptides	Mean ± SD (8 h)	Mean ± SD (24 h)
23.6 kDa heat shock protein	Q96331	HSP23.6	13.45	20.00	5	-	4.68 ± 0.43
15.7 kDa heat shock protein	Q9FHQ3	HSP15.7	3.92	15.33	2	-	3.72 ± 0.29
12-oxophytodienoate reductase 3	Q9FUP0	OPR3	4.67	5.37	2	-	2.56 ± 0.10
Catalase-3	Q42547	CAT3	17.43	22.56	9	-	2.38 ± 0.22
Plasma membrane-associated cation-binding protein 1	Q96262	PCAP1	7.70	27.11	5	2.22 ± 0.26	-
V-type proton ATPase subunit G1	O82628	VHA-G1	7.10	43.64	7	1.82 ± 0.41	0.56 ± 0.05
Catalase-2	P25819	CAT2	8.99	10.98	5	-	1.98 ± 0.45
Glycerate dehydrogenase	Q9C9W5	HRP1	8.10	11.14	4	-	1.83 ± 0.32
Oxygen-evolving enhancer protein 3-1	Q9XFT3	PSBQ1	10.88	20.98	5	-	1.81 ± 0.23
Homocysteine methyltransferase	O50008	CIMS	5.14	5.62	3	-	1.79 ± 0.02
Peroxisomal (S)-2-hydroxy-acid oxidase	Q9LRR9	GLO1	10.35	19.62	6	-	1.71 ± 0.35
NADH dehydrogenase [ubiquinone] flavoprotein 2	O22769	At4g02580	8.40	18.04	4	-	1.67 ± 0.15
Adenylate kinase 1	O82514	ADK1	15.95	36.99	7	1.55 ± 0.14	-
Patellin-1	Q56WK6	PATL1	7.97	4.89	3	1.50 ± 0.10	-
Glutathione S-transferase F2	P46422	GSTF2	8.99	10.98	3	-	1.49 ± 0.22
Heat shock protein 90-3	P51818	HSP90-3	8.10	11.14	4	-	1.47 ± 0.10
Cytochrome b-c1 complex subunit 7-1	Q9SUU5	QCR7-1	6.44	40.16	5	1.42 ± 0.01	-
Mitochondrial outer membrane protein porin 2	Q9FJX3	VDAC2	7.10	11.96	6	1.40 ± 0.05	-
Probable NADH dehydrogenase [ubiquinone] 1 alpha subcomplex subunit 5	Q9FLX7	At5g52840	13.58	37.87	7	1.39 ± 0.04	-
Malate dehydrogenase	Q9ZP05	At5g09660	7.84	14.41	4	1.38 ± 0.17	-
Mitochondrial outer membrane protein porin 1	Q9SRH5	VDAC1	7.97	28.62	6	1.36 ± 0.05	-
NADH dehydrogenase [ubiquinone] iron-sulfur protein 1	Q9FGI6	EMB1467	10.13	7.89	6	-	1.36 ± 0.15
Probable arginase	Q9ZPF5	At4g08870	20.60	43.90	12	1.35 ± 0.08	1.35 ± 0.08
ATP synthase subunit d	Q9FT52	At3g52300	19.84	54.17	9	1.35 ± 0.05	1.35 ± 0.05
V-type proton ATPase subunit B3	Q8W4E2	VHA-B3	21.24	23.82	10	1.35 ± 0.06	0.67 ± 0.01
Glyceraldehyde-3-phosphate dehydrogenase	P25858	GAPC	10.50	21.60	6	1.34 ± 0.05	1.39 ± 0.01
Mitochondrial outer membrane protein porin 3	Q9SMX3	VDAC3	16.93	35.77	13	1.34 ± 0.07	-
Formate dehydrogenase	Q9S7E4	FDH1	14.52	23.18	9	-	1.33 ± 0.04
Mitochondrial-processing peptidase subunit beta	Q42290	At3g02090	34.46	41.81	22	1.32 ± 0.00	-
Dihydrolipoyl dehydrogenase 1	Q9M5K3	LPD1	4.61	5.72	2	-	1.31 ± 0.08
Probable ATP synthase 24 kDa subunit	Q9SJ12	At2g21870	19.94	27.08	8	-	1.31 ± 0.15
Succinate dehydrogenase [ubiquinone] flavoprotein subunit 1	O82663	SDH1-1	8.86	8.52	4	-	1.31 ± 0.07
60S ribosomal protein L13-1	P41127	RPL13B	5.68	19.90	3	1.31 ± 0.07	-
CBS domain-containing protein	Q9LEV3	CBSX3	5.47	18.45	3	1.30 ± 0.02	1.45 ± 0.06
Ribulose bisphosphate carboxylase large chain	O03042	rbcL	32.95	31.94	19	0.76 ± 0.02	-
60S ribosomal protein L17-2	P51413	RPL17B	5.32	16.57	2	0.75 ± 0.06	-
UPF0603 protein	Q9ZVL6	At1g54780	18.42	40.70	8	0.75 ± 0.07	-
Chlorophyll a-b binding protein CP26	Q9XF89	LHCB5	16.85	27.14	13	0.73 ± 0.04	-
Apocytochrome f	P56771	petA	40.06	53.44	23	0.73 ± 0.02	-
Calcium sensing receptor	Q9FN48	CAS	6.61	12.14	4	-	0.73 ± 0.03
Photosystem I reaction center subunit III	Q9SHE8	PSAF	13.04	23.08	7	-	0.73 ± 0.02
50S ribosomal protein L16	P56793	rpl16	4.15	20.74	2	-	0.70 ± 0.00
V-type proton ATPase catalytic subunit A	O23654	VHA-A	22.44	17.50	11	-	0.70 ± 0.02
Glycine dehydrogenase [decarboxylating] 2	Q94B78	At4g33010	14.38	9.35	7	0.70 ± 0.06	-
Geranylgeranyl diphosphate reductase1	Q9CA67	CHLP	12.10	12.42	6	0.70 ± 0.04	-
Chlorophyll a-b binding protein CP29.1	Q07473	LHCB4.1	23.47	28.62	16	0.70 ± 0.01	-
Cytochrome b6-f complex iron-sulfur subunit	Q9ZR03	petC	10.58	23.58	7	0.69 ± 0.01	-
Photosystem II CP47 chlorophyll apoprotein	P56777	psbB	30.58	22.64	25	0.69 ± 0.03	0.54 ± 0.01
Clathrin heavy chain 1	Q0WNJ6	CHC1	2.96	0.82	1	0.69 ± 0.03	-
Protein TIC110	Q8LPR9	TIC110	4.49	1.77	2	-	0.66 ± 0.06
40S ribosomal protein S9-1	Q9LXG1	RPS9B	18.15	34.85	8	-	0.66 ± 0.02
Lipoxygenase 2	P38418	LOX2	11.99	16.29	12	0.66 ± 0.03	-
ATP synthase subunit b	P56759	atpF	19.29	44.57	14	0.65 ± 0.04	-
ADP,ATP carrier protein 1	P31167	AAC1	14.85	19.95	7	0.64 ± 0.05	-
Photosystem II 22 kDa protein	Q9XF91	PSBS	3.60	6.04	2	0.62 ± 0.04	-
L-ascorbate peroxidase T	Q42593	APXT	2.60	9.16	2	0.62 ± 0.02	-
Photosystem Q(B) protein	P83755	psbA	8.81	17.28	13	0.62 ± 0.04	-
60S ribosomal protein L18a-2	P51418	RPL18AB	3.25	5.06	2	-	0.62 ± 0.04
60S ribosomal protein L21-2	Q9FDZ9	RPL21E	6.81	11.59	3	-	0.62 ± 0.07
V-type proton ATPase subunit E1	Q39258	VHA-E1	4.40	11.74	3	-	0.61 ± 0.01
Photosystem II CP43 chlorophyll apoprotein	P56778	psbC	23.73	28.33	14	0.61 ± 0.03	0.60 ± 0.03
Thylakoid lumenal 16.5 kDa protein	O22773	At4g02530	6.90	18.06	4	0.60 ± 0.09	-
Photosystem I P700 chlorophyll a apoprotein A2	P56767	psaB	13.90	10.49	7	-	0.59 ± 0.01
Photosystem II D2 protein	P56761	psbD	16.40	22.38	15	0.56 ± 0.02	0.54 ± 0.04
Photosystem I P700 chlorophyll a apoprotein A1	P56766	psaA	10.60	6.00	5	0.55 ± 0.05	0.58 ± 0.05
ATP synthase subunit alpha	P56757	atpA	43.10	37.87	25	-	0.52 ± 0.05
Oxygen-evolving enhancer protein 1-1	P23321	PSBO1	40.9	58.73	25	0.49 ± 0.04	0.72 ± 0.01
ATP synthase gamma chain 1	Q01908	ATPC1	22.92	26.54	10	-	0.46 ± 0.03
ATP synthase epsilon chain	P09468	atpE	10.29	25.00	5	-	0.41 ± 0.02
ATP synthase subunit beta	P19366	atpB	59.44	64.26	39	-	0.41 ± 0.02
Aquaporin PIP2-1	P43286	PIP2-1	8.23	10.45	4	-	0.38 ± 0.07

**Table 2:** Classification of the differentially expressed proteins in response to OTA using the STRING database.

Protein Name	UniProtKB Accession	Mean ± SD (8 h)	Mean ± SD (24 h)	Protein Name	UniProtKB Accession	Mean ± SD (8 h)	Mean ± SD (24 h)
**Group 1 Photosynthesis**				**Group 3 ATP synthase**			
Calcium sensing receptor	Q9FN48		0.73 ± 0.03	V-type proton ATPase catalytic subunit A	O23654		0.67 ± 0.02
Photosystem II CP47 chlorophyll apoprotein	P56777	0.69 ± 0.03	0.54 ± 0.01	ATP synthase subunit alpha	P56757		0.52 ± 0.05
Apocytochrome f	P56771	0.73 ± 0.02		ATP synthase epsilon chain	P09468		0.41 ± 0.02
Cytochrome b6-f complex iron-sulfur subunit	Q9ZR03	0.69 ± 0.01		ATP synthase gamma chain 1	Q01908		0.46 ± 0.03
L-ascorbate peroxidase T	Q42593	0.62 ± 0.02		ATP synthase subunit b	P56759	0.65 ± 0.04	
Photosystem I P700 chlorophyll apoprotein A1	P56766	0.55 ± 0.05		ATP synthase subunit beta	P19366		0.41 ± 0.02
Photosystem I P700 chlorophyll apoprotein A2	P56767		0.59 ± 0.01	V-type proton ATPase subunit B3	Q8W4E2	1.35 ± 0.06	
Photosystem II CP43 chlorophyll apoprotein	P56778		0.60 ± 0.03	V-type proton ATPase subunit E1	Q39258		0.61 ± 0.01
Chlorophyll a-b binding protein CP29.1	Q07473	0.69 ± 0.01		ATP synthase subunit d	Q9FT52	1.35 ± 0.05	
Chlorophyll a-b binding protein CP26	Q9XF89	0.73 ± 0.04		V-type proton ATPase subunit G1	O82628	1.82 ± 0.41	0.56 ± 0.05
Geranylgeranyl diphosphate reductase1	Q9CA67	0.70 ± 0.04		**Group 4 Translation, ribosome biogenesis**			
Photosystem Q(B) protein	P83755	0.62 ± 0.04		60S ribosomal protein L13-1	P41127	1.31 ± 0.07	
UPF0603 protein	Q9ZVL6	0.75 ± 0.07		60S ribosomal protein L17-2	P51413	0.75 ± 0.06	
Thylakoid lumenal 16.5 kDa protein	O22773	0.60 ± 0.09		50S ribosomal protein L16	P56793		0.70 ± 0.00
Photosystem II 22 kDa protein	Q9XF91	0.62 ± 0.04		40S ribosomal protein S9-1	Q9LXG1		0.66 ± 0.02
Photosystem II D2 protein	P56761	0.56 ± 0.02	0.54 ± 0.04	60S ribosomal protein L21-2	Q9FDZ9		0.62 ± 0.07
Oxygen-evolving enhancer protein 3-1	Q9XFT3		1.81 ± 0.23	60S ribosomal protein L18a-2	P51418		0.62 ± 0.04
Oxygen-evolving enhancer protein 1-1	P23321	0.49 ± 0.04	0.72 ± 0.01	**Group 5 Mitochondrial transport protein**			
Photosystem I reaction center subunit III	Q9SHE8		0.73 ± 0.02	Probable ATP synthase 24 kDa subunit	Q9SJ12		1.31 ± 0.15
Lipoxygenase 2	P38418	0.66 ± 0.03		Mitochondrial-processing peptidase subunit beta	Q42290	1.32 ± 0.00	
**Group 2 Mitochondrial electron transport**				Mitochondrial outer membrane protein porin 3	Q9SMX3	1.34 ± 0.07	
NADH dehydrogenase	Q9FGI6		1.36 ± 0.15	Mitochondrial outer membrane protein porin 1	Q9SRH5	1.36 ± 0.05	
Cytochrome b-c1 complex subunit 7-1	Q9SUU5	1.42 ± 0.01		Mitochondrial outer membrane protein porin 2	Q9FJX3	1.40 ± 0.05	
Probable NADH dehydrogenase	Q9FLX7	1.39 ± 0.04		ADP,ATP carrier protein 1	P31167	0.64 ± 0.05	
Succinate dehydrogenase flavoprotein subunit 1	O82663		1.31 ± 0.07				
NADH dehydrogenase flavoprotein 2	O22769		1.67 ± 0.15				

**Table 3:** Expression profile of genes related to nucleic acid metabolism that responded to OTA.

Gene Category	ID	Gene Description	Fold Change (log_2_)
DNA	AT3G18500	Endonuclease/exonuclease/phosphatase family protein	4.0 (2.0)
Endonuclease	AT3G10010	Demeter-like 2 (DML2)	3.7 (1.9)
	AT5G17540	Bifunctional nuclease I (BFN1)	90.5 (6.5)
	AT1G59720	Chlororespiratory reduction 28 (CRR28)	59.7 (5.9)
	AT1G53250	Endonuclease	59.7 (5.9)
	AT2G21800	Essential meiotic endonuclease 1A (EME1A)	59.7 (5.9)
	AT1G73875	Endonuclease/exonuclease/phosphatase family protein	2.5 (1.4)
	AT3G21530	Endonuclease/exonuclease/phosphatase family protein	2.4 (1.3)
	AT1G68290	Endonuclease 2 (ENDO2)	2.3 (1.2)
DNA replicate	AT2G07690	Minichromosome maintenance family protein (MCM5)	−2.8 (−1.5)
	AT1G67630	DNA polymerase alpha 2 (POLA2)	−3.0 (−1.6)
	AT1G67320	DNA primase	−4.0 (−2.0)
	AT4G02060	Minichromosome maintenance family protein (MCM7)	−4.6 (−2.2)
	AT1G10590	DNA-binding protein-related	2.2 (1.1)
DNA repair	AT1G10590	DNA-binding protein-related	2.2 (1.1)
	AT4G17020	DNA repair related	2.1 (1.1)
RNA	AT5G60040	DNA-directed RNA polymerase (NPRC1)	10.3 (3.4)
polymerase	AT1G54250	DNA-directed RNA polymerase II, core complex (NPR8A)	6.1 (2.6)
	AT3G52090	DNA-directed RNA polymerase II, core complex (NP11)	3.4 (1.8)
	AT5G60040	DNA-directed RNA polymerase (NPRC1)	10.3 (3.4)
RNA	AT2G36530	Copper ion binding/ phosphopyruvate hydratase (LOS2)	2.9 (1.6)
degradation	AT1G17980	Nucleotidyltransferase family protein	2.5 (1.3)

**Table 4:** Expression profile of genes related to mitochondria that responded to OTA.

Gene Category	ID	Gene Description	Fold Change (log_2_)
TCA	AT1G24180	Pyruvate dehydrogenase E1a-like subunit (IAR4)	5.9 (2.6)
	AT1G01090	Pyruvate dehydrogenase E1 alpha	2.4 (1.2)
	AT2G44350	ATP citrate synthase (ATCS)	3.8 (1.9)
	AT3G58750	Citrate synthase 2 (CSY2)	2.0 (1.0)
	AT2G17130	Isocitrate dehydrogenase subunit 2 (IDH2)	9.1 (3.2)
	AT3G09810	Isocitrate dehydrogenase, putative	5.2 (2.4)
	AT5G03290	Isocitrate dehydrogenase, putative	4.7 (2.4)
	AT4G26910	2-oxoacid dehydrogenase family protein	3.8 (1.9)
	AT2G05710	Aconitate hydratase (Aconitase)	4.0 (2.0)
	AT5G23250	Succinyl-CoA ligase, putative	2.2 (1.3)
	AT5G40650,	Succinate dehydrogenase (SDH2-2)	2.5 (1.3)
	AT2G47510	Fumarase 1 (FUM1)	3.4 (1.8)
	AT5G09660	NAD-malate dehydrogenase 2 (PMDH2)	−2.3 (−1.2)
Oxidative phosphorylation			
Complex I	AT3G12260	Complex 1 family protein	7.6 (2.9)
	AT5 G47890	NADH-ubiquinone oxidoreductase B8 subunit, putative	9.7 (3.3)
	AT1G79010	NADH-ubiquinone oxidoreductase 23 kDa subunit	2.4 (1.3)
	AT4G05020	NAD(P)H dehydrogenase B2 (NDB2)	3.6 (1.9)
	AT3G06310	NADH-ubiquinone oxidoreductase 19 kDa subunit (NDUFA8)	−2.2 (−1.2)
Complex II	AT5G40650	Succinate dehydrogenase (SDH2-2)	2.5 (1.3)
Complex III	AT5G25450	Ubiquinol-cytochrome C reductase complex 14 kDa protein	4.7 (2.2)
Complex IV	AT3G15352	Cytochrome c oxidase, Copper chaperone (COX17)	3.7 (1.9)
	AT2G44520	Cytochrome c oxidase 10 (COX10)	3.0 (1.6)
	AT1G22450	Cytochrome c oxidase 6B (COX6B)	2.1 (1.1)
ATPase	AT2G33040	ATP synthase gamma chain	2.6 (1.1)
(Complex V)	AT4G30190	Hydrogen-exporting ATPase	2.2 (1.2)
	AT4G02620	ATPase subunit F family protein	5.9 (2.6)
	AT4G11150	ATP synthase subunit E1 (TUF)	4.8 (2.3)
	AT3G28715	H^+^-transporting two-sector ATPase, putative	3.7 (1.9)
	AT2G18960	H^+^-ATPase 1 (AHA1)	2.8 (1.5)
	AT2G21410	Vacuolar proton ATPase A2 (VHA-A2)	2.8 (1.5)
	AT2G28520	Vacuolar proton ATPase A1 (VHA-A1)	2.7 (1.4)
	AT5G62670	H^+^-ATPase 11 (AHA11)	−3.0 (−1.6)

## Supplementary Materials

The following are available online at www.mdpi.com/2072-6651/9/5/167/s1, Figure S1: The protein-protein interaction networks were analyzed using the STRING database.
