# Supplementary material for: iTRAQ Mitoproteome Analysis Reveals Mechanisms of Programmed Cell Death in Arabidopsis thaliana Induced by Ochratoxin A
Source: Toxins (Basel). 2017 May 18;9(5):167. doi: 10.3390/toxins9050167 (PMC5450715; doi:10.3390/toxins9050167)
Supplement: Supplementary file 1 [file toxins-09-00167-s001.pdf]

Supplementary Materials: iTRAQ Mitoproteome Analysis Reveals Mechanisms of Programmed Cell Death in *Arabidopsis Thaliana* Induced by Ochratoxin A

Yan Wang, Xiaoli Peng, Zhuojun Yang, Weiwei Zhao, Wentao Xu, Junran Hao, Weihong Wu, Xiao Li Shen, Yunbo Luo and Kunlun Huang

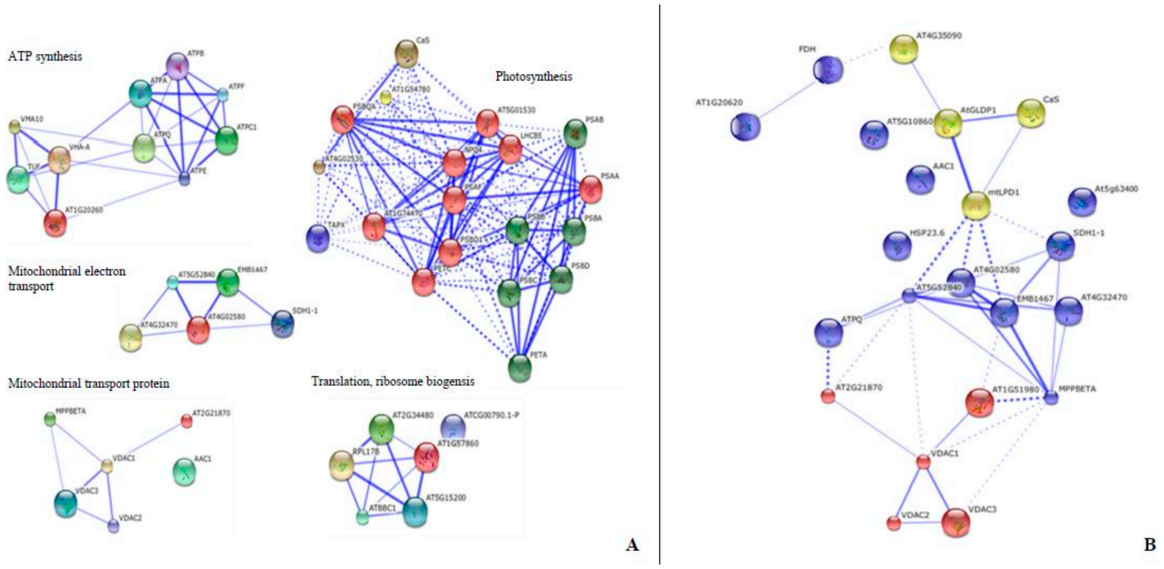

Figure S1. The protein–protein interaction networks were analyzed using the STRING database. (**A**) Most of differentially expressed proteins identified. (**B**)The differentially expressed proteins located in mitochondria. The representative networks were profiled according to GO and PANTHER biological processes, molecular functions or KEGG pathways, and were integrated with the STRING database. The interactions among proteins that are strongly supported by previous work are linked by blue lines. The gene names of corresponding proteins are displayed in the networks. The red balls represent proteins that participated in the mitochondrial membrane signal recognition and membrane transport, the central blue balls represent proteins involved in the mitochondrial electron transport chain coupling oxidation and phosphorylation, and the yellow balls represent proteins that participated in amino acid metabolism and played arole asa calcium-sensing receptor.
